# Similarities and differences between European guidelines for the management of postmenopausal osteoporosis

**DOI:** 10.1007/s11657-024-01441-z

**Published:** 2024-09-05

**Authors:** Bernard Cortet, Núria Guañabens, Maria Luisa Brandi, Heide Siggelkow

**Affiliations:** 1 Department of Rheumatology and ULR 4490 (MabLab), University-Hospital of Lille, Lille, France; 2 Rheumatology Department, Hospital Clinic, IDIBAPS, University of Barcelona, C. de Villarroel 170, 08036 Barcelona, Spain; 3 Italian Bone Disease Research Foundation, Fondazione Italiana Ricerca Sulle Malattie Dell’Osso (FIRMO), Florence, Italy; 4 Department of Trauma, Orthopedic and Reconstructive Surgery, University Medical Center Göttingen, Robert-Koch-Str, Göttingen, Germany; 5 MVZ Endokrinologikum Göttingen, Von-Siebold-Str, Göttingen, Germany

**Keywords:** European guidelines, Postmenopausal osteoporosis, Fracture risk

## Abstract

**Summary:**

We conducted a review of 10 national guidelines from five EU countries to identify similarities or differences in recommendations for the management of patients with osteoporosis. We found general alignment of key recommendations; however, there are notable differences, largely attributed to country-specific approaches to risk assessment and reimbursement conditions.

**Introduction:**

The classification of fracture risk is critical for informing treatment decisions for post-menopausal osteoporosis. The aim of this review was to summarise 10 national guidelines from five European countries, with a focus on identifying similarities or differences in recommendations for the management of patients with osteoporosis.

**Methods:**

We summarised the European Society for Clinical and Economic Aspects of Osteoporosis, Osteoarthritis and Musculoskeletal Disease-International Osteoporosis Foundation guidelines and reviewed guidelines from France, Germany, Italy, Spain and the UK.

**Results:**

The approach to risk assessment differed across the guidelines. In France, and Spain, risk assessment was based on DXA scans and presence of prior fractures, whereas UK, German and Italian guidelines recommended use of a validated risk tool. These differences led to distinct definitions of very high and high-risk patients. Guidelines aligned in recommending antiresorptive and anabolic agents as pharmacologic options for the management of osteoporosis, with sequential treatment recommended. There was agreement that patients at high or very high risk of fracture or with severe osteoporosis should receive anabolic agents first, followed by antiresorptive drugs. Variations were identified in recommendations for follow up of patients on anti-osteoporosis therapies. Reimbursement conditions in each country were a key difference identified.

**Conclusions:**

Criteria for risk assessment of fractures differ across European guidelines which may impact treatment and access to anabolic agents. Harmonisation across EU guidelines may help identify patients eligible for treatment and impact treatment uptake. However, country-specific reimbursement and prescribing processes may present a challenge to achieving a consistent approach across Europe.

## Introduction

Osteoporosis is a skeletal disorder characterised by a decline in bone density and micro-architectonical deterioration of bone tissue leading to reduced mechanical strength and increased propensity to fracture [1–6]. The SCOPE 2021 summary report estimated that there are more than 23 million women and men at high risk of osteoporotic fractures in the EU [1, 7]. In fact, the annual number of osteoporotic fractures in the EU was calculated to be 4.28 million in 2019, and this is projected to increase to 5.34 million in 2034 [1].

In 2019, on average, the treatment gap, defined as the rate of women who exceed the intervention threshold but do not receive treatment, in the EU27 + 2 countries was 71%. Furthermore, 15 million women eligible for osteoporosis therapy remained untreated [7]. The reasons for the treatment gap are currently unclear but may, in part, be due to a decline in bone mineral density (BMD) testing due to reimbursement issues. In addition, in recent years, the uptake of treatments for osteoporosis, particularly bisphosphonates, has declined, due to concerns around safety, including risk of osteonecrosis of the jaw and/or atypical subtrochanteric femur fractures [1]. Importantly, a low awareness of the significant burden of osteoporosis on health and quality of life, by healthcare professionals, may further contribute to the treatment gap [8].

Together, these findings indicate there is a need for clear and consistent guidance across the EU countries in order to ensure consistent and high levels of osteoporosis care.

### Current guidelines for osteoporosis management in Europe

In 2020, the European Society for Clinical and Economic Aspects of Osteoporosis and Osteoarthritis-International Osteoporosis Foundation (ESCEO-IOF) updated the 2019 guidance for the diagnosis and management of osteoporosis in postmenopausal women [9, 10]. In addition, individual EU countries have their own guidelines for the management of postmenopausal osteoporosis. One of the challenges is a lack of harmonisation among national guidelines which could lead to different standards of care across the countries in the EU. It is important to note that healthcare authorities and insurance companies take into account the reimbursement conditions for diagnostic and treatment modalities, which may differ between European countries, thus potentially impacting clinical practice [11]. The aim of this review was to compare and contrast national guidelines and the ESCEO-IOF guidelines for the management of osteoporosis to identify any differences that may exist.

## Materials and methods

This review will discuss the guidelines that are currently applied in the following countries: France, Germany, Italy, Spain, UK. The guidelines were selected by the authors as the most relevant and frequently used for the management of osteoporosis in five major countries of the EU. However, not all European guidelines were included; only those with English translations, from countries with a large number of inhabitants and/or from the countries represented by the authors, were included. Those included are shown in Table 1. In the UK, NICE is currently consulting on, and developing, updated guidance for osteoporosis risk assessment, treatment and fragility fracture prevention, and this is due in 2025 [12]. This will replace the current CG146 which does not contain any recommendations for treatment and fracture prevention.

**Table 1:** National guidelines included in the review

Country	Society	Title	Language	Date	Ref
France	Bone Task Force of the French Society for rheumatology (SFR) and of the Osteoporosis Research and Information Group (GRIO),	2018 update of French recommendations on the management of postmenopausal osteoporosis	English	2018	[13]
Germany	Dachverband Osteologie	Prophylaxe, Diagnostik und Therapie der Osteoporose (in German/English)	German/ English	2023	[14, 24]
Italy	Sistema Nazionale per le Linee Guida	Diagnosi, stratificazione del rischio e continuità assistenziale delle fratture da fragilità 2021 (in Italian)	Italian	2021	[33]
	Sistema Nazionale per le Linee Guida	Executive summary: Italian guidelines for diagnosis, risk stratification, and care continuity of fragility fractures 2021	English	2023	[20]
	Inter-Society Commission for Osteoporosis	Guidelines for the management of osteoporosis and fragility fractures	English	2019	[15]
Spain	Spanish Society for Bone and Mineral Metabolism Investigation (SEIOMM)	Clinical practice guideline of postmenopausal, glucocorticoid-induced and male osteoporosis: 2022 update	English	2022	[16, 17]
UK	National Institute for Health and Care Excellence	Clinical Guideline CG146 Osteoporosis: assessing the risk of fragility fracture^a^	English	2012, last updated 2017	[5]
	National Institute for Health and Care Excellence	Guideline NG23 menopause: diagnosis and management	English	2015, last updated 2019	[34]
	National Institute for Health and Care Excellence	Clinical knowledge summary osteoporosis—prevention of fragility fractures	English	2023	[2]
	National Osteoporosis Guideline Group (NOGG) UK	Clinical guideline for the prevention and treatment of osteoporosis	English	2021	[6]

^a^NICE is currently developing updated guidance for osteoporosis risk assessment, treatment and fragility fracture prevention, to be delivered by 2025[11]

### Fracture risk assessment

#### Risk assessment

All national guidelines studied in this review applied the same definition for the diagnosis of osteoporosis [6, 9, 13–17]. Moreover, all guidelines recommended that patients should be initially assessed for clinical risk factors for osteoporosis, including the presence of prior fragility fractures [6, 9, 13–17]. However, considerable variation was observed in the approach towards the assessment of fracture risk across the guidelines studied.

Several risk engines exist for calculating the future risk of fracture, including the Garvan fracture risk calculator, QFracture, FRAX®, De-FRA and DVO fracture risk calculators [6, 9, 13–17]. The ESCEO-IOF guidelines recommend that postmenopausal women with a prior fragility fracture should be treated without further assessment, although BMD measurement and incorporation into the FRAX calculation is sometimes appropriate, particularly in younger postmenopausal women. A country-specific FRAX® is used to assess fracture probability in postmenopausal women who have risk factors for fracture [9, 10]. FRAX® is calibrated to those countries where the epidemiology of fracture and death is known (including all countries studied in this review, except Spain) [9, 10].

The limitations of FRAX® are widely recognised. The FRAX® assessment takes no account of dose-responses for risk factors such as cigarette exposure, alcohol intake or number of prior fractures, fracture site or corticosteroid dose or duration [9, 18]. It is also accepted that FRAX® underestimates fracture risk in patients with diabetes mellitus [19]. In addition, it does not include a history of falls, a significant risk factor for fracture [9]. Consequently, some countries, for example, Germany and Italy, have developed their own risk assessment tools [14, 15, 20]. Alternatively, to address some limitations, simple arithmetic adjustments have been proposed that can be applied to conventional FRAX® estimates to adjust the probability assessments [9]. More recently, the FRAXplus® tool has been developed to overcome some of these concerns; however, there is a charge associated with its use, and it is currently undergoing testing and not yet recommended for clinical purposes [18, 21].

In line with ESCEO-IOF [9, 10], following the assessment of clinical risk factors, the UK guidelines recommend the use of FRAX® [6] or FRAX®/QFracture [2, 22] to predict the 10-year absolute risk of MOF in those with a clinical risk factor for fragility fracture. Patients with a high risk of fragility fractures should receive treatment and undergo BMD measurement to inform treatment and provide a baseline for BMD monitoring [6]. Patients at very high risk of fragility fractures should also receive treatment and undergo BMD measurements with the same rationale, but should also be considered for referral to a specialist [6]. NICE also notes that BMD should not routinely be used to assess fracture risk without prior assessment using FRAX® or QFracture [2, 22]. However, it is recommended to measure BMD to assess fracture risk in patients < 40 years who have a major risk factor such as a history of multiple fragility fractures, MOF or current or recent use of high-dose oral or high-dose systemic glucocorticoids [2].

The Spanish SEIOMM 2022 guidelines recommended assessment of BMD via DXA when clinical risk factors are strongly associated with osteoporosis or fractures [16, 17]. A combination of clinical data, including fragility fractures, especially vertebral and hip fracture, and DXA is recommended to assess fracture risk. FRAX® has had limited uptake in Spain, and its adaptation to the national epidemiology of fractures has been inadequate leading to underestimation of the risk of MOFs [16, 17]. Patients determined to be at very high or high risk of fracture, the latter defined by ≥ 1 fragility fracture, a *T*-score <  − 2.5 in the lumbar spine, femoral neck or total hip, or a *T*-score <  − 2.0 with strong clinical risk factors for osteoporosis should receive treatment [16, 17].

In Germany, BMD measurement using DXA at the lumbar spine and proximal femur on both sides is a core part of the basic diagnostics for risk of osteoporosis and fractures in postmenopausal women and men above 50 years of age with a risk factor for osteoporosis and in those for whom treatment is being considered [14]. If the result of a DXA BMD measurement does not provide a sufficient basis for medical decision-making, an alternative measurement method for fracture risk assessment can be considered [14]. The absolute 3-year fracture risk is calculated using the DVO fracture risk calculator, which is based on German reference data and includes the imminent fracture risk [14, 23]; the 2023 DVO guidelines recommend that the 3-year fracture risk estimate replaces that of the 10-year fracture risk estimate and provides a table to support conversion between the two during the transition period. Treatment is recommended for patients classed as at very high or high risk of fracture [24].

The French guidelines take a similar approach to Germany and Spain, with BMD measurements indicated for patients with clinical risk factors for osteoporosis, such as prior fragility fracture, systemic glucocorticoid therapy, diseases known to cause secondary osteoporosis, menopause < 40 years or BMI < 19 kg/m^2^ [13]. The FRAX® tool is recommended to calculate the 10-year probability of hip fracture and MOF; however, the guidelines note that the FRAX® tool is not useful when there is a clear indication to start osteoporosis therapy, for example, where there is a history of severe fracture, as defined by Bliuc et al. [25], or a *T*-score ≤  − 3 at the lumbar spine and total hip/femoral neck [13]. When using the FRAX® tool, the cut off for treatment is the value in same-age women with a history of fracture (risk of repeat fracture) as recommended by the ESCEO-IOF guidelines [13].

In Italy, the use of a validated risk tool is recommended for patients with clinical risk factors for osteoporosis [20]. National algorithms have been developed based on FRAX®, including the Italian FRAX-derived version (DeFRA) and FRActure Health Search (FRA-HS) tools [15, 20], with DeFRA showing better performance than FRAX® for women and diabetics [20]. The risk of fracture should always be obtained by integrating densitometric data with clinical risk factors [15]. However, similar to the UK, patients with a very high risk of fragility fractures, such as those with a history of previous osteoporotic fractures, or chronic glucocorticoid therapy may proceed to drug therapy without BMD densitometric measurements [15]. Patients with a high or imminent risk of (re)fracture are recommended to receive pharmacological treatment [20].

#### Definitions of very high and high risk of osteoporotic fracture

The definition of very high and high risk of fracture varied considerably across the guidelines studied (Table 2), in line with the different risk tools and approaches to risk stratification used in the different countries. Guidelines for France, Italy, Spain and the UK generally defined patients at very high risk as those with severe fractures and/or very low BMD scores [6, 13, 16, 17, 20]; however, the number of vertebral (or vertebral or hip) fractures required to meet the definition varied from ≥ 1 to ≥ 3 [6, 13, 16, 17, 20], and the BMD *T*-score required to meet the definition was − 3.5 T in patients without prior fractures [6, 16, 17] and ranged from − 4 to − 1.5 T in patients with prior fractures [13, 16, 17, 20]. The UK NOGG guidelines also stipulated a threshold that characterises patients at high and very high risk of fracture using FRAX® probabilities. Here, very high risk was identified as a FRAX-based fracture probability that exceeds the intervention threshold by 60% [6].

**Table 2:** Risk assessment

	France	Germany	Italy		Spain	UK		Europe
	Briot et al. (2018)	DVO (2023)	Corrao et al. (2023)	Nuti et al. (2019)	Riancho et al. (2022)	CG146 (2017)	NOGG (2021)	ESCEO-IOF
Fracture risk tool	FRAX® based on national validation and calibration	DVO risk model	De-FRA/De-FRAcalc79, or FRA-HS	DeFRA, FRAX®, or Fra-HS	Combination of clinical data and DXA	FRAX® or QFracture	FRAX®	Country-specific FRAX®
BMD DXA measurement	Indicated in all patients with clinical risk factors	Indicated in all patients with clinical risk factors	Measure risk with De-FRA first. DXA indicated in patients with intermediate risk	Measure risk with De-FRA first. DXA indicated in patients with intermediate risk	Indicated in all patients with clinical risk factors	Measure risk with FRAX® first. DXA indicated in patients with intermediate risk	Measure risk with FRAX® first. DXA indicated in patients with intermediate risk/to guide treatment	Measure risk with FRAX® first. DXA Indicated in patients with intermediate risk
Intervention threshold (IT)	Severe fracture: BMD *T*-score ≤ − 1.0 Non-severe fracture: BMD *T*-score ≤ − 2.0 Patients without fractures: BMD *T*-score ≤ − 3.0 (lumbar spine and/or hip)	DVO calc 3-year fracture risk of ≥ 3%	-	IT based on FRAX® or DeFRA	≥ 1 fragility fractures; or BMD *T*-score < − 2.5; or BMD *T*-score < − 2.0 together with factors strongly associated with fracture risk	IT based on FRAX- 10-year probability of MOF	IT based on FRAX- 10-year probability of MOF	FRAX®-based 10-year probability (%) of MOF
Definition of very high-risk	• ≥ 2 vertebral fractures • BMD ≤ − 3 T + severe fracture	• ≥ 10% 3-year absolute fracture risk calculated by DVO risk calculator	• ≥ 3 vertebral or hip fractures • ≥ 1 vertebral or hip fractures + BMD ≤ − 4	-	• ≥ 2 vertebral fractures or equivalent^a^ • 1 spine or hip fracture + BMD < − 3.0 T or • BMD < − 3.5 T	-	• FRAX-based fracture probability exceeds IF by 60% • Recent vertebral fracture [≤ 2 years] • ≥ 2 vertebral fractures • BMD ≤ − 3.5 T • Treatment with high dose glucocorticoids	
Definition of high-risk	• ≥ 1 severe fragility fracture and BMD ≤ − 1 T) • ≥ 1 non-severe fracture and BMD ≤ − 2 T) • BMD ≤ − 3 T)	• ≥ 5% 3-year absolute fracture risk calculated by DVO risk calculator	• Fragility fracture	• Femoral BMD < − 2.5 T • Prior vertebral fractures and femoral BMD < − 2.0 T	• Fragility fracture • BMD < − 2.5 T • Low BMD + high risk factors^b^	• Above upper age limit for 10-year absolute fracture risk, defined by FRAX® or QFracture	• Above upper age limit for 10-year absolute fracture risk, defined by FRAX®	• An age-specific fracture probability equivalent to same-age women with a prior fragility fracture

*BMD* bone mineral density, *DXA* dual energy X-ray absorptiometry, *IT* intervention threshold, *MOF* major osteoporotic fracture, *TBS* Trabecular bone score

^a^For example, vertebral and hip fracture

^b^Especially if *T* ≤  − 2 and factors strongly associated with fracture risks, such as hypogonadism, early menopause or treatment with glucocorticoids or sex hormone antagonists

^c^Indicated in postmenopausal women if they have a history of ≥ 4-cm height loss, kyphosis, recent/current long-term oral glucocorticoid therapy, *T*-score ≤  − 2.5 or in cases of acute-onset back pain with risk factors for osteoporosis

#### Biochemical assessment of fracture risk

The ESCEO-IOF guidelines state that there is a modest but significant association between bone turnover markers and the future risk of fracture and adds that there are efforts underway to harmonise markers of bone turnover which may lead to their use in fracture risk prediction [9]. However, the German, Italian and French guidelines state that bone remodelling parameters should not routinely be part of the basic diagnosis of osteoporosis [13–15]. The Spanish SEIOMM guidelines recommend that bone turnover markers, together with other risk factors, can aid in identifying patients at a higher risk of fracture, and, above all, help in the early assessment of response to treatment [16, 17]. The Italian, French and UK guidelines commented that biochemical indices of skeletal turnover can support treatment monitoring [6, 13, 15].

#### Trabecular bone score and vertebral fracture assessment

In line with ESCEO-IOF guidelines [9, 10], the majority of guidelines assessed agreed that the trabecular bone score (TBS) may be considered as an adjunct to BMD and FRAX® and can improve the prediction of fracture risk [6, 14–16]. Moreover, most guidelines recommended vertebral fracture assessment (VFA) may be carried out for certain, high-risk, patient groups [6, 9, 13–17] (Table 2).

### Pharmacological interventions for osteoporosis

The choice of drug for the management of post-menopausal osteoporosis depends on factors such as individual fracture risk, type of fracture, potential drug-related adverse events, intolerances and/or contraindications, patient preference and cost [6, 13, 14, 24]. Antiresorptive therapies available for post-menopausal osteoporosis management include bisphosphonates, denosumab, selective oestrogen receptor modulators (SERMs) and hormone replacement therapy (HRT). Anabolic agents include teriparatide and romosozumab, which is a dual-action drug [2, 6, 9, 13–17, 20, 24]; an additional anabolic agent, abaloparatide, a synthetic peptide analogue of parathyroid hormone-related protein was approved in Europe in October 2022 for the treatment of osteoporosis in postmenopausal women at increased risk of fracture.

European guidelines recommended the use of oral bisphosphonates in high-risk patients (Table 3) [6, 10, 16, 24]. In France, bisphosphonates are reimbursed for patients with severe non-vertebral fractures (i.e., fractures at the hip, proximal humerus, pelvis, femoral shaft, distal femur, ribcage involving at least three ribs, and proximal tibia), vertebral fractures, non-severe fractures (wrist and other sites) and in patients without fractures but with low BMD and risk factors for osteoporosis [13]. The UK guidelines suggested use of oral bisphosphonates for patients at high risk of fracture [6]. The Spanish and ESCEO-IOF 2020 guidelines aligned with this, but also suggested use of other antiresorptive drugs [10, 16, 17].

**Table 3:** Pharmacological management of osteoporosis

	UK		France	Spain	Italy		Germany	Europe
	NICE CKS (2023)^a^	NOGG (2021)^b^	Briot et al. (2018)^c^	SEIOMM (2022)^d^	Corrao et al. (2023)^e^	Nuti et al. (2019)	DVO (2023)^f^	ESCEO-IOF^g^
Recommended treatments								
Oral bisphosphonates	Y	Y	Y (SNVF,VF,NSF)	Y (HFR/VF,NVF,HF)		Y	Y	Y
IV bisphosphonates	Y	Y	Y (SNVF,VF,NSF)	Y (HFR/VF,NVF,HF)		Y	Y	Y
Denosumab	Y	Y	Y (SNVF,VF,NSF)	Y (HFR/VF,NVF,HF)			Y	Y
Teriparatide	Y	Y (VHFR)	Y (VF)	Y (VHFR)	Y (HFR)	Y (HFR)	Y (VHFR)	Y (HFR/VF)
Romosozumab		Y (VHFR)		Y (VHFR)	Y (HFR)		Y (VHFR)	
Duration of treatment								
Oral bisphosphonates	3–5 years HFR (7–10 years)	≥ 5 years HFR/VHFR (≥ 10 years)	5 years	5 years or (10 years)		5 years (HFR ≤ 10 years)	Alendronate (RCT: 3 years) (ET:10 years) Risedronate (RCT: 3–5 years) (ET: 7 years) Ibandronate (RCT: 3 years) (ET: 5 years)	5 years
IV bisphosphonates		≥ 3 years HFR/VHFR (≥ 6 years)	3 years (zoledronate)	3 years or (6 years) (zoledronate)		3 years or HFR ≤ 6 years (zoledronate)	Zoledronate (RCT: 3 years) (ET: 6 years) Ibandronate (RCT: 2 years) (ET:5 years)	3 years (zoledronate)
Denosumab			3 years	5–10 years			RCT: 3 years ET: 6–10 years	
Teriparatide		24 months	18 months	24 months		24 months	24 months/lifetime	
Romosozumab		12 months		12 months			12 months/cycle	

*ET* extension trials, *HF* hip fracture, *HFR* high fracture risk, *LFR* low fracture risk, *MFR* moderate fracture risk, *NSF* non-severe fractures, *NVF* non-vertebral fracture, *RCT* randomised controlled trials, *SNVF* severe non-vertebral fractures, *SO* severe osteoporosis, *VHFR* very high fracture risk, *VF* vertebral fracture

^a^NICE CKS: If oral bisphosphonates not tolerated or contraindicated, consider other options as outlined in the table, via a specialist. Longer treatment up to 7 years with risedronate and up to 10 years with alendronate advised in patients with high fracture risk

^b^NOGG, 2021: Consider IV zoledronate first line following hip fracture, if not tolerated, consider denosumab or ibandronate. Consider teriparatide or romosozumab first line if very high fracture risk or second-line if bisphosphonate intolerance

^c^Briot et al. (2018): Parenteral drugs (zoledronate and denosumab) may be preferentially used in patients with hip fractures, very low BMD values, comorbidities, memory impairments, poor adherence and polypharmacy. Consider zoledronate first line for patients with hip fracture and teriparatide first line in patients with two prevalent vertebral fractures. For patients without fractures, where treatment is indicated, treatment options are those as listed for non-severe fractures

^d^SEIOMM, 2022: Parenteral zoledronate or denosumab recommended if intolerance, adherence, polypharmacy issues, comorbidities or in patients > 75 years of age. Alendronate may be given for up to 10 years and zoledronate for up to 6 years

^e^Corrao et al. (2023): Recommend anabolic agents followed by antiresorptive therapy in patients at high or imminent risk of (re)fracture, in patients who experienced a fragility fracture

^f^DVO, 2023: Drugs listed in the table are approved in Germany. Oral bisphosphonates are usually first line on the basis of cost. Denosumab or IV bisphosphonates may be appropriate in other circumstances. Teriparatide and romosozumab are superior to oral bisphosphonates for fracture-related endpoints. Bone anabolic agents are recommended if absolute fracture risk is above the bone anabolic threshold and should be considered if absolute fracture risk is above treatment threshold but below the bone anabolic threshold. If, in an individual case, the increase in BMD is of particular importance, consider denosumab over other antiresorptive agents

^g^ESCEO-IOF: Oral bisphosphonates may be used as initial treatments, with alendronate considered first line based on cost and parenteral zoledronate or denosumab considered as alternatives if there are intolerance issues or contraindications. Teriparatide is preferentially recommended for patients at high risk of fractures, with a strong recommendation for use in patients with vertebral fractures

The German DVO guidelines advised to consider bone anabolic agents for high-risk individuals who fall above the treatment threshold, and noted that, where the primary intended outcome is fracture-related, teriparatide or romosozumab should be considered in preference to antiresorptive drugs as both have shown superiority to oral bisphosphonates for fracture-related endpoints [14]. However, beyond this, there is no preference for use of one anti-osteoporosis drug over another, since there are no head-to-head studies comparing fracture endpoint data. Since German healthcare regulations require physicians to prescribe the most cost-effective therapy, oral bisphosphonates are generally selected first line [14, 24]. This aligns with the UK, where oral bisphosphonates, namely alendronate and risedronate, or intravenous zoledronate are recommended first line [2, 6] due to cost-effectiveness [6]. The French guidance did not mention use of ibandronate for osteoporosis management, as reimbursement for ibandronate has not been provided in France since 2011 [13].

ESCEO-IOF 2019 guidelines suggested that the presence of intolerances or contraindications to use of oral bisphosphonates may necessitate use of parenteral therapy with zoledronate or denosumab [9]. This recommendation appeared consistently across the European guidelines [2, 6, 9, 13, 16, 17, 20, 24]. However, the UK NOGG and French guidelines specifically outlined use of zoledronate as first-line treatment following a hip fracture, with alternatives such as denosumab if not tolerated [6, 13], while the Spanish SEIOMM guidelines suggest that injectable antiresorptive agents are preferable for patients over 75 years of age [16, 17]. The German DVO guidelines preferentially suggested use of denosumab if the increase in BMD is of specific importance in any individual case [14, 24]. For patients at high risk of fracture, the UK NOGG guidance recommended denosumab or other antiresorptive drugs where oral bisphosphonates are not tolerated or unsuitable [6]. This recommendation aligned with the Spanish SEIOMM and the DVO guidelines which also recommended denosumab as an option for high-risk individuals [14, 16, 17]. In France, denosumab is reimbursed for the treatment of postmenopausal osteoporosis in women at increased risk of fracture, as second-line therapy after bisphosphonates.

SERMs, specifically raloxifene, were recommended by French, Spanish and ESCEO-IOF guidelines for certain patients or as an alternative to IV bisphosphonates or denosumab in patients with contraindications or intolerance to oral bisphosphonates [9, 13, 16, 17]. European guidelines limited the use of HRT to younger postmenopausal women, with use restricted to symptomatic postmenopausal women in Germany, due to the associated risk of cardiovascular events and breast cancer. HRT is a particular option for those with contraindications to other osteoporosis therapies [2, 6, 9, 13–17, 20].

Teriparatide and romosozumab are anabolic drugs, with romosozumab having a dual mode of action, both increasing bone formation and decreasing bone resorption [26]. Both agents are recommended as options in Germany, Italy, France, the UK and Spain for patients at high risk [24], imminent risk of (re)fracture [20], patients with severe fractures and very low BMD [13] and very high risk of fracture [6, 14, 16, 17]. They are also suggested as alternative options where there are intolerances or contraindications to the use of oral bisphosphonates in the UK [6]. Teriparatide is recommended in the ESCEO-IOF 2019 and Italian guidelines for patients at high risk of fracture and in the ESCEO-IOF 2020 guidelines for patients at very high risk, followed by antiresorptive therapies [9, 10, 15]. The Italian guidelines also recommend use in patients unresponsive to antiresorptive drugs [9, 15].

In the French, Spanish SEIOMM, UK and ESCEO-IOF guidelines, there is a recommendation for first-line use of teriparatide in patients with vertebral fractures [6, 9, 13, 16, 17]. The French, Italian and ESCEO-IOF 2019 guidelines did not mention romosozumab, although this is likely due to these guidelines pre-dating the availability of romosozumab [9, 13, 15, 27]. Moreover, romosozumab is not reimbursed in France.

Abaloparatide is listed in the ESCEO-IOF guidelines as an intervention used in the management of postmenopausal osteoporosis [10]. The DVO 2023 guidelines note that the German Osteoporosis Group had not evaluated abaloparatide as it had not been approved and was not available in Europe and Germany at the time of conducting the systematic literature review, although an amendment is to be expected [14, 24]. The Spanish guidelines commented that abaloparatide was not available in Spain at the time they were written [16].

Over the lifetime of a patient with osteoporosis, it is accepted that more than one medication will be needed and treatment typically involves sequential therapy [9, 10]. The duration of treatment for many anti-osteoporosis therapies is limited (see also “Duration of treatment,” below). Consequently, the guidelines agreed that sequential treatment strategies are required [2, 6, 9, 13–17, 20]. In most countries, switching from an antiresorptive to an anabolic treatment is not a first-line choice, unless there is confirmed failure of the antiresorptive drug. This is because previous use of a bisphosphonate can slightly reduce the BMD gained with teriparatide [18, 19]. Moreover, the effect of the anabolic appears to depend on the specific properties of the antiresorptive drug used before switching [28]. Therefore, where indicated, the Spanish, Italian, German, UK and ESCEO-IOF guidelines all suggested preferential initial use of an anabolic [6, 9, 10, 14, 16, 17, 20].

There is a risk of bone loss once anabolic treatments are stopped [20]. As such, the ESCEO-IOF French, German, Spanish and UK guidelines added that teriparatide should be followed immediately by treatment with an antiresorptive agent (bisphosphonate or denosumab) [6, 9, 10, 13, 16, 17, 20, 24]. The ESCEO-IOF guidelines further stated that the benefits of teriparatide are maintained if denosumab is prescribed as soon as possible after stopping the anabolic drug [9]. The Italian guidelines noted that the anabolic-antiresorptive sequence is effective for secondary prevention of fragility fracture but added there is still some uncertainty about the strength of the evidence [20].

Teriparatide should not be initiated as the only treatment in the months following denosumab, given the risk of accelerated bone loss, especially at the distal radius and hip [16, 17, 29]. However, it should be noted that limited evidence is available to support sequential therapy options as head-to-head comparisons are not available for most registered treatments, combinations or sequences [28].

Conflicting results have been observed in studies investigating the effect of combination therapy for osteoporosis; this was not extensively discussed across the European guidelines and not routinely recommended as per the Spanish guidelines. However, on an individual basis, in severe cases at very high or high risk of hip fracture, the Spanish guidelines did suggest that combined use of teriparatide with denosumab or zoledronate may be considered [16, 17]. This aligned with the ESCEO-IOF guidelines which indicated that combined use of antiresorptive and bone forming agents may be beneficial for hip outcomes [9] and the German guidelines, which suggests combination therapy with teriparatide and parenteral antiresorptive agent for patients with a very high imminent risk of fracture [14].

#### Follow-up care

The follow-up care recommendations for patients on anti-osteoporosis therapies varied across the guidelines studied [2, 6, 9, 13–17, 20]. Monitoring for adherence, treatment tolerability and adverse effects was highlighted across the European, UK, French, Italian, Spanish and German guidelines [2, 6, 9, 13–17], with assessment for symptoms of atypical fractures [2] and acute spinal and back pain also recommended in France and Germany [13, 14]. In conjunction with this, height checks were advised as height loss may indicate vertebral fractures [13, 14]. The French guidelines also recommended counselling patients on the risk of osteonecrosis of the jaw and atypical femoral fractures with denosumab and bisphosphonate therapy during follow-up [13], with a dental assessment to support care in France and Germany [13, 24]. Follow-up reassessment of risk factors for falls and determination of a falls history was recommended in the French and German DVO 2023 guidelines [13, 14], with a UK recommendation to review medication that may predispose to falls and fractures [6].

Fracture risk reassessment was highlighted in the UK, German and ESCEO-IOF guidelines [6, 9, 14], with a recheck of BMD via a DXA scan advised in France, Germany, Italy and Spain [4, 13, 14, 16, 17] and FRAX + DXA in the UK [6]. The French guidelines recommended follow-up DXA scans 2–3 years after oral bisphosphonate therapy, 3 years following zoledronate or denosumab and after 18 months of teriparatide treatment [13]. The UK, Italian, French and Spanish guidelines recommended a treat-to-target approach, using BMD as a surrogate marker [6, 13, 15, 16]. Whilst it was noted that the BMD target may vary with age and the site at greatest risk for fracture [13], guidelines from Spain, France and Italy were aligned in recommending a target *T*-score of ≥  − 2.5 or − 2 at the femur for a severe fracture in a patient with a very low femoral BMD value [13, 15, 16]. The UK guidelines recommended treatment should continue until the patient is below the intervention threshold and the *T*-Score is >  − 2.5 [6].

To assess response to oral treatment by an inhibitor of bone resorption and to inform the dosage 3–6 months after initiation, monitoring of bone turnover markers using serum carboxy-terminal collagen crosslinks (CTX) was recommended in France [13]. The German and Spanish guidelines stated that surrogate parameters of bone metabolism (bone density and bone turnover markers) can be monitored to assess the effect of therapy [13, 16, 17].

There were variations across the European guidelines between recommended timings for following up patients post-fracture or those taking anti-osteoporosis medicines. The UK NOGG guidelines support the Royal Osteoporosis Society recommendation of follow-up within 16 weeks and 52 weeks post-fracture [6]. The DVO 2023 guidelines recommended that patients should initially be reviewed every 3–6 months after initiating specific treatments in order to assess the effect of therapy and take into account data for the prediction of fractures [14]. The UK guidelines recommended reviewing anti-osteoporosis treatment after 3–5 years in patients taking bisphosphonates or denosumab [6]. In Spain, patients treated with bisphosphonates should be evaluated after 3 (zoledronate) or 5 years (oral bisphosphonates) of treatment; patients treated with denosumab should be evaluated after 5–10 years of treatment [16, 17]. This guidance was replicated in the ESCEO-IOF 2019 document for oral and intravenous bisphosphonates (review after 5 years and 3 years of treatment respectively) [9]. The French guidance suggested reviewing BMD 2–3 years after treatment initiation or whenever a change in treatment is considered; however, the guidelines indicate the duration of initial treatment with oral bisphosphonates and zoledronic acid should be 5 and 3 years, respectively [13].

#### Duration of treatment

European guidelines noted that duration of treatment will depend on the patient’s risk of fracture, and the patient’s preferences regarding treatment should be taken into account [6, 14, 16, 17]. Guidelines recommended treatment durations of oral bisphosphonates of 5–10 years, with 10 years advised where longer-term management is indicated, and recommended 3–6 years for intravenous bisphosphonates, with the increased duration indicated for longer-term treatment. This was consistent across the UK guidelines, and guidance in France, Spain and Italy [6, 13, 15–17]. The German guidance aligned with these and stated that continuation of a specific treatment beyond 3–5 years is justified in the case of a high 10-year fracture risk [14]. The DVO 2023 guidance outlined durations as per randomised controlled trials, extension trials and legal regulations, suggesting 3–10 years oral bisphosphonate use, 3 years for IV zoledronate and 2 years for IV ibandronate [14, 24]. The ESCEO-IOF 2019 and French guidelines also recommended oral bisphosphonates for 5 years and intravenous bisphosphonates for 3 years for a first sequence; however, the overall duration of treatment can be longer [9, 13].

The Spanish SEIOMM 2022 guidelines commented that denosumab can be administered for 5–10 years [16, 17]. There was no limitation for the duration of denosumab in the French, Italian or German guidelines [4, 13–15, 20]. At the end of the periods, the decision to stop or continue treatment should be determined based on the residual fracture risk [13]. ESCEO-IOF 2019 guidelines added that there is little evidence to guide decision-making beyond 10 years of treatment, and patient management should be evaluated on an individual basis [9]. Since there is a risk of rebound increase in bone turnover upon stopping denosumab [30], Italian, German, ESCEO-IOF 2019, UK, French and Spanish guidelines advised follow-up treatment with an antiresorptive drug when denosumab is stopped [6, 9, 13–17]. The French guidelines also note that oral or injectable bisphosphonate therapy should be given for 6–12 months when denosumab is stopped as there is evidence that bisphosphonates may prevent bone loss after denosumab discontinuation [13]. The UK and Spanish guidelines aligned in their recommendation to initiate intravenous zoledronate post-last denosumab dose and to use measurements of bone turnover markers to guide timing of further zoledronate doses [6, 16, 17]. Where monitoring is not possible, further zoledronate doses should be given 6 months [6] and 6–12 months [16, 17] following the initial zoledronate infusion [6, 16, 17]. The Spanish guidelines further suggest that when denosumab is used for less than 2.5 years, alendronate may be used instead of zoledronate, according to the recommendations of the Position Statement by the European Calcified Tissue Society [31].

The use of teriparatide and abaloparatide is time-limited due to a theoretical risk of osteosarcoma which was observed with near-lifetime/high-dose treatment in rodents [14, 32]. However, this has not been seen in humans during post-marketing surveillance [14]. Teriparatide is recommended for 24 months per lifetime in the UK, Germany, Italy and Spain [6, 14–17, 24]. In contrast, the French guidelines recommended 18-month treatment with teriparatide on the basis of reimbursement availability; however, it can be prescribed for 24 months without reimbursement for the last 6 months [13]. Use of abaloparatide is limited to 18 months [32]. The recommended romosozumab duration is 12 months per treatment cycle in the UK, Germany and Spain [6, 14, 16, 17, 24].

#### Drug treatment holidays

Drug treatment holidays were only recommended in the context of previous bisphosphonate treatment and did not apply to denosumab or anabolics which should be followed by bisphosphonate or denosumab treatment, as described above. For high-risk patients, treatment continuation was advised in the UK [2]. This is aligned with French guidance which suggested further treatment where the hip *T*-score is <  − 2.5 after the recommended initial treatment period of oral or intravenous bisphosphonates or denosumab [13] and the Spanish guidance, which suggested continuing therapy if the femoral neck *T*-score is <  − 2.5 [16, 17]. In Spain, treatment should also be continued if fragility fractures occur during treatment, 3–5 years prior to evaluation [16, 17]. The UK (NICE) guidelines aligned in suggesting continuing treatment in patients with a prior hip or vertebral fracture [2]; in Spain, some experts recommend continuing treatment if the patient has a history of hip or vertebral fracture on a case-by-case basis [16, 17]. In Italy, guidelines advised that in patients at high risk of (re)fracture, treatment should be continued unless serious adverse events occur [20], for up to 10 years in those receiving oral bisphosphonates and up to 6 years if prescribed zoledronate [15]. German DVO 2023 guidance aligned with other European countries, suggesting bisphosphonate treatment continuation or switch to an alternative where the fracture risk remains high, above the DVO treatment threshold [14].

Consideration should be given to stopping treatment if the repeat DXA scan reveals a *T*-score >  − 2.5 in the UK [2]. In France, drug holidays should be considered in patients with no fractures during treatment, no new risk factors nor significantly reduced BMD at the spine or hip and in patients with a history of severe fracture, a femoral *T*-score ≥  − 2.5 or − 2 [13]. In Germany, similar advice regarding considering a pause in bisphosphonate treatment was recommended for patients whose fracture risk drops below the DVO treatment threshold [14]. In Spain, in the absence of the factors described above which would necessitate treatment continuation, temporary withdrawal of bisphosphonate treatment could be considered [16, 17].

Following bisphosphonate treatment, a suggested hiatus of 12–24 months was recommended by Italian guidelines and 12–36 months by Spanish guidelines [15–17]. In Spain, for denosumab, temporary interruptions in treatment were not advised [16, 17].

#### Restarting osteoporosis treatment

After pausing drug therapy for osteoporosis, it is necessary to reassess fracture risk [6]. ESCEO-IOF and UK NOGG guidelines also advised reassessing fracture risk after a new fracture [6, 9]. UK guidelines recommended restarting treatment in the event of a new fracture and if there is a relapse from suppressed bone turnover and/or a reduction in the BMD [6]. The UK and French guidelines generally aligned in their recommendation that fracture risk should be re-assessed 18 months–3 years (UK) or 2 years (France) post-treatment discontinuation, depending on the type of bisphosphonate used [6, 13]. Conversely, the German DVO 2023 guidance suggested monitoring bone turnover markers and/or BMD via a DXA scan during a drug holiday, with a view to initiate treatment if the fracture risk increases above the DVO treatment threshold [14]. In Spain, the need to reinstate antiosteoporosis treatment should be assessed periodically.

#### Breakthrough fracture while on osteoporosis treatment

In the UK, it is recommended to assess for non-adherence to medication and investigate for secondary causes of osteoporosis if new fractures occur while on treatment [2, 6]. Referral for specialist advice on drug treatment should be considered if secondary causes are excluded [2]. Spanish guidance aligned with this, suggesting alternative treatment in the event of breakthrough fractures [16, 17].

## Conclusions

Our review identified general alignment in the management of osteoporosis, related to fracture risk–specific treatment choices, treatment duration and sequences across the country-specific guidelines from the EU. Key differences emerged in recommendations for patient risk assessment, which can lead to differences in access to anabolic agents for suitable candidates and pharmacological treatment options. This may also in part be related to reimbursement and national availability, which appears to be a major factor leading to differences in osteoporosis management across the European countries studied in this review.

Moving forwards, the European Commission should play a key role in driving forwards harmonisation of guidelines where possible, by establishing standardised practices for osteoporosis diagnosis and management across Europe. Lastly, the variations highlighted within this guideline review should serve as supportive tools for guiding management choices. However, these should not supersede clinical judgement, which should come first and foremost when delivering care to individual patients.

## Data Availability

Data can be made available upon request.
